# Supplementary material for: Preventing iatrogenic HCV infection: A quantitative risk assessment based on observational data in an Egyptian hospital
Source: PLOS Glob Public Health. 2024 Feb 15;4(2):e0002821. doi: 10.1371/journal.pgph.0002821 (PMC10868760; doi:10.1371/journal.pgph.0002821)
Supplement: S1 Table — (DOCX) [file pgph.0002821.s002.docx]

**Table S1.** Characteristics of each group found after sequence analysis.

| *Characteristic* | *Group 1, N = 356* | *Group 2, N = 54* | *Group 3, N = 14* | *Group 4, N = 76* | *p-value* | *Overall, N = 500* |
| --- | --- | --- | --- | --- | --- | --- |
| ***Gender*** |  |  |  |  | *0·6* |  |
| *Female* | *151 (42%)* | *19 (35%)* | *6 (43%)* | *36 (47%)* |  | *212 (42%)* |
| *Male* | *205 (58%)* | *35 (65%)* | *8 (57%)* | *40 (53%)* |  | *288 (58%)* |
| ***Age*** | *43 (30, 58)* | *42 (29, 57)* | *64 (46, 67)* | *53 (37, 64)* | ***0·006*** | *45 (30, 60)* |
| ***Education level*** |  |  |  |  | *0·058* |  |
| *No formal education* | *156 (44%)* | *16 (30%)* | *8 (57%)* | *44 (58%)* |  | *224 (45%)* |
| *Primary or preparatory school* | *53 (15%)* | *10 (19%)* | *2 (14%)* | *11 (14%)* |  | *76 (15%)* |
| *Secondary school or higher* | *147 (41%)* | *28 (52%)* | *4 (29%)* | *21 (28%)* |  | *200 (40%)* |
| ***Marital status*** |  |  |  |  | *0·6* |  |
| *Single* | *54 (15%)* | *8 (15%)* | *2 (14%)* | *7 (9·2%)* |  | *71 (14%)* |
| *Maried* | *268 (75%)* | *38 (70%)* | *10 (71%)* | *58 (76%)* |  | *374 (75%)* |
| *Widow* | *27 (7·6%)* | *5 (9·3%)* | *2 (14%)* | *7 (9·2%)* |  | *41 (8·2%)* |
| *Divorced* | *7 (2·0%)* | *3 (5·6%)* | *0 (0%)* | *4 (5·3%)* |  | *14 (2·8%)* |
| ***Localization*** |  |  |  |  | *0·12* |  |
| *Cairo* | *244 (69%)* | *37 (69%)* | *8 (57%)* | *42 (55%)* |  | *331 (66%)* |
| *Other governate* | *110 (31%)* | *17 (31%)* | *6 (43%)* | *34 (45%)* |  | *167 (34%)* |
| *Unknown* | *2* | *0* | *0* | *0* |  | *2* |
| ***Patient Hospitalized before*** | *258 (72%)* | *40 (74%)* | *12 (86%)* | *66 (88%)* | ***0·031*** | *376 (75%)* |
| *Unknown* | *0* | *0* | *0* | *1* |  | *1* |
| ***Source of admission*** |  |  |  |  | *0·082* |  |
| *Outpatient clinic* | *151 (42%)* | *32 (59%)* | *5 (36%)* | *38 (50%)* |  | *226 (45%)* |
| *Emergency room* | *205 (58%)* | *22 (41%)* | *9 (64%)* | *38 (50%)* |  | *274 (55%)* |
| ***Hospital at recruitment*** |  |  |  |  | ***<0·001*** |  |
| *Surgery hospital* | *219 (62%)* | *54 (100%)* | *1 (7·1%)* | *0 (0%)* |  | *274 (55%)* |
| *Internal medicine hospital* | *137 (38%)* | *0 (0%)* | *13 (93%)* | *76 (100%)* |  | *226 (45%)* |
| ***Status at the end of following*** |  |  |  |  | ***<0·001*** |  |
| *Deceased* | *0 (0%)* | *0 (0%)* | *5 (36%)* | *0 (0%)* |  | *5 (1·0%)* |
| *Discharged* | *356 (100%)* | *54 (100%)* | *9 (64%)* | *76 (100%)* |  | *495 (99%)* |
| ***Average estimated risk of HVC infection (%)*** | *0·04% (0·02%, 0·07%)* | *0·07% (0·05%, 0·11%)* | *0·47% (0·08%, 0·82%)* | *0·12% (0·03%, 0·30%)* | ***<0·001*** | *0·04% (0·03%, 0·09%)* |
| ***Duration of hospitalization (days)*** | *2·3 (1·7, 3·3)* | *6·4 (5·4, 9·0)* | *20·4 (17·5, 23·2)* | *8·7 (7·2, 10·9)* | ***<0·001*** | *3·1 (1·9, 5·8)* |
| ***Number of procedures*** | *6 (4, 8)* | *14 (9, 23)* | *43 (15, 77)* | *12 (5, 23)* | ***<0·001*** | *7 (4, 11)* |
| **Reason for hospitalisation** |  |  |  |  | ***<0·001*** |  |
| General surgery | *152 (43%)* | *26 (48%)* | *2 (14%)* | *1 (1·3%)* |  | *181 (36%)* |
| Special surgery | *54 (15%)* | *23 (43%)* | *2 (14%)* | *22 (29%)* |  | *101 (20%)* |
| General IM | *30 (8·4%)* | *0 (0%)* | *2 (14%)* | *12 (16%)* |  | *44 (8·8%)* |
| Special IM | *78 (22%)* | *4 (7·4%)* | *6 (43%)* | *33 (43%)* |  | *121 (24%)* |
| Liver/ GIT complaint | *42 (12%)* | *1 (1·9%)* | *2 (14%)* | *8 (11%)* |  | *53 (11%)* |
| *n (%); Median (IQR)* | | | | | | |
