# Supplementary material for: Preventing iatrogenic HCV infection: A quantitative risk assessment based on observational data in an Egyptian hospital
Source: PLOS Glob Public Health. 2024 Feb 15;4(2):e0002821. doi: 10.1371/journal.pgph.0002821 (PMC10868760; doi:10.1371/journal.pgph.0002821)
Supplement: S2 Table — (DOCX) [file pgph.0002821.s003.docx]

**Table S2.** Summary of the impact of patient and ward-focused strategies on the risk of HCV infection during hospitalization.

| **Intervention** | **Average risk reduction**  **(Std. error)** | **Average risk**  **(Std. error)** | **Number of patients concerned by intervention (% of total)** | **Wards concerned for the ward-focused scenario (Number)** |
| --- | --- | --- | --- | --- |
| *Patient-focused*  *(Model-based selection)* | 80.12%  (9.14x10^-4^) | 0.023%  (8.46x10^-5^) | 200 (40%) | - |
|  | 73.01%  (9.00x10^-4^) | 0.031%  (2.71x10^-4^) | 150 (30%) | - |
|  | 52.51%  (1.22x10^-3^) | 0.058%  (1.86x10^-4^) | 100 (20%) | - |
|  | 28.35%  (8.57x10^-3^) | 0.078%  (4.37x10^-4^) | 50 (10%) |  |
| *Patient-focused*  *(Random selection)* | 63.39%  (9.29x10^-3^) | 0.042%  (1.03x10^-3^) | 200 (40%) | - |
|  | 49.56%  (1.31x10^-3^) | 0.058%  (1.29x10^-3^) | 150 (30%) | - |
|  | 34.95%  (1.45x10^-2^) | 0.075%  (1.75x10^-3^) | 100 (20%) | - |
|  | 18.51%  (1.56x10^-2^) | 0.087%  (2.05x10^-3^) | 50 (10%) | - |
| *Ward-focused* | 76.93%  (4.48x10^-4^) | 0.027%  (8.26x10^-5^) | 203 (40.6%) | Rheumatology, Tropical medicine, ER ICU, GIT and endoscopy, Cardiology,Neurology,Immunology,Urosurgery,Ophtalmology (9) |
|  | 62.97%  (7.47x10^-4^) | 0.043%  (7.83x10^-5^) | 159 (31.8%) | Rheumatology, Tropical medicine, ER ICU, GIT and endoscopy, Cardiology (5) |
|  | 56.02%  (1.07x10^-3^) | 0.051%  (1.21x10^-4^) | 82(16.4%) | Rheumatology, Tropical medicine, ER ICU, GIT and endoscopy (4) |
|  | 30.00%  (2.98x10^-3^) | 0.077%  (3.24x10^-4^) | 53(10.6%) | Rheumatology, Tropical medicine, ER ICU (3) |
