# Supplementary material for: Preventing iatrogenic HCV infection: A quantitative risk assessment based on observational data in an Egyptian hospital
Source: PLOS Glob Public Health. 2024 Feb 15;4(2):e0002821. doi: 10.1371/journal.pgph.0002821 (PMC10868760; doi:10.1371/journal.pgph.0002821)
Supplement: S3 Table — (DOCX) [file pgph.0002821.s004.docx]

**Table S3.** Sensitivity analysis for the cut-off value of the risk considered in the logistic regression.

| **Quantile proba. for overall risk in baseline scenario** | **Cut-off value for the risk (%)** | **AUC [CI95%]** | **Specificity [CI95%]** | **Sensitivity [CI95%]** | **Precision [CI95%]** | **Informedness** |
| --- | --- | --- | --- | --- | --- | --- |
| 0.60 | 0.056% | *0.76 [0.69-0.83]* | 0.62[0.54-0.71] | 0.69[0.58-0.78] | 0.55[0.45-0.64] | 0.31 |
| 0.65 | 0.066% | *0.80[0.73-0.87]* | 0.76[0.68-0.83] | 0.70[0.58-0.79] | 0.61[0.5-0.71] | 0.46 |
| 0.70 | 0.076% | *0.82[0.75-0.89]* | 0.72[0.64-0.79] | 0.73[0.61-0.83] | 0.53[0.42-0.63] | 0.45 |
| 0.75 | 0.096% | *0.79[0.71-0.87]* | 0.73[0.65-0.79] | 0.68[0.54-0.79] | 0.45[0.35-0.57] | 0.41 |
| 0.80 | 0.12% | *0.78[0.69-0.87]* | 0.71[0.64-0.78] | 0.70[0.55-0.82] | 0.38[0.28-0.49] | 0.41 |
| 0.85 | 0.16% | *0.77[0.67-0.87]* | 0.75[-0.68-0.81] | 0.67[0.39-0.73] | 0.29[0.19-0.41] | 0.32 |
| 0.90 | 0.25% | *0.82[0.7-0.94]* | 0.64[0.57-0.71] | 0.90[0.7-0.97] | 0.22[0.14-0.32] | 0.54 |
| 0.95 | 0.49% | *0.72[0.54-0.9]* | 0.75[0.68-0.8] | 0.60[0.31-0.83] | 0.11[0.05-0.22] | 0.35 |
