# Supplementary figures and images for: Preventing iatrogenic HCV infection: A quantitative risk assessment based on observational data in an Egyptian hospital
Source: PLOS Glob Public Health. 2024 Feb 15;4(2):e0002821. doi: 10.1371/journal.pgph.0002821 (PMC10868760; doi:10.1371/journal.pgph.0002821)

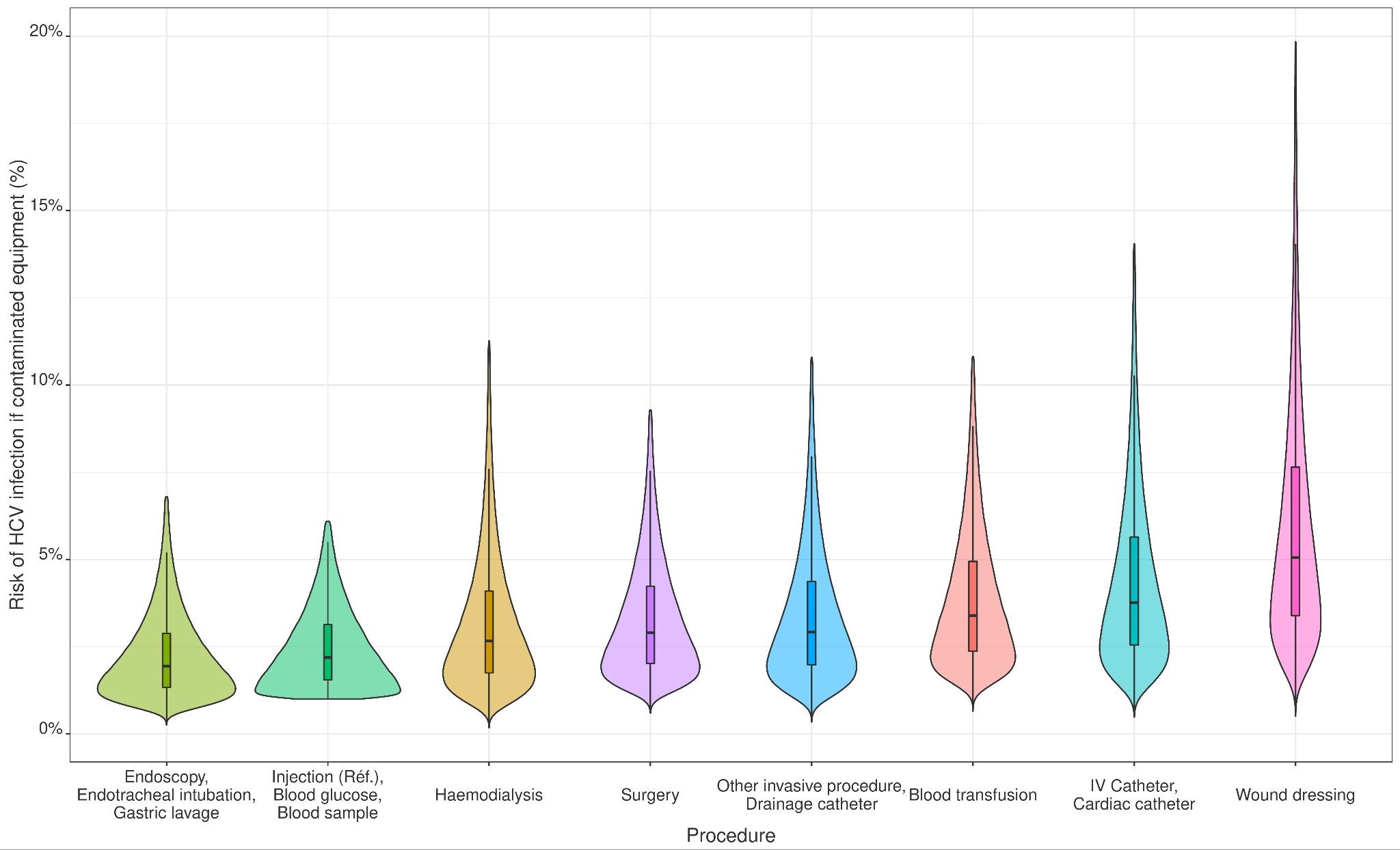

Supplement: S1 Fig — (JPG) [file pgph.0002821.s005.jpg]

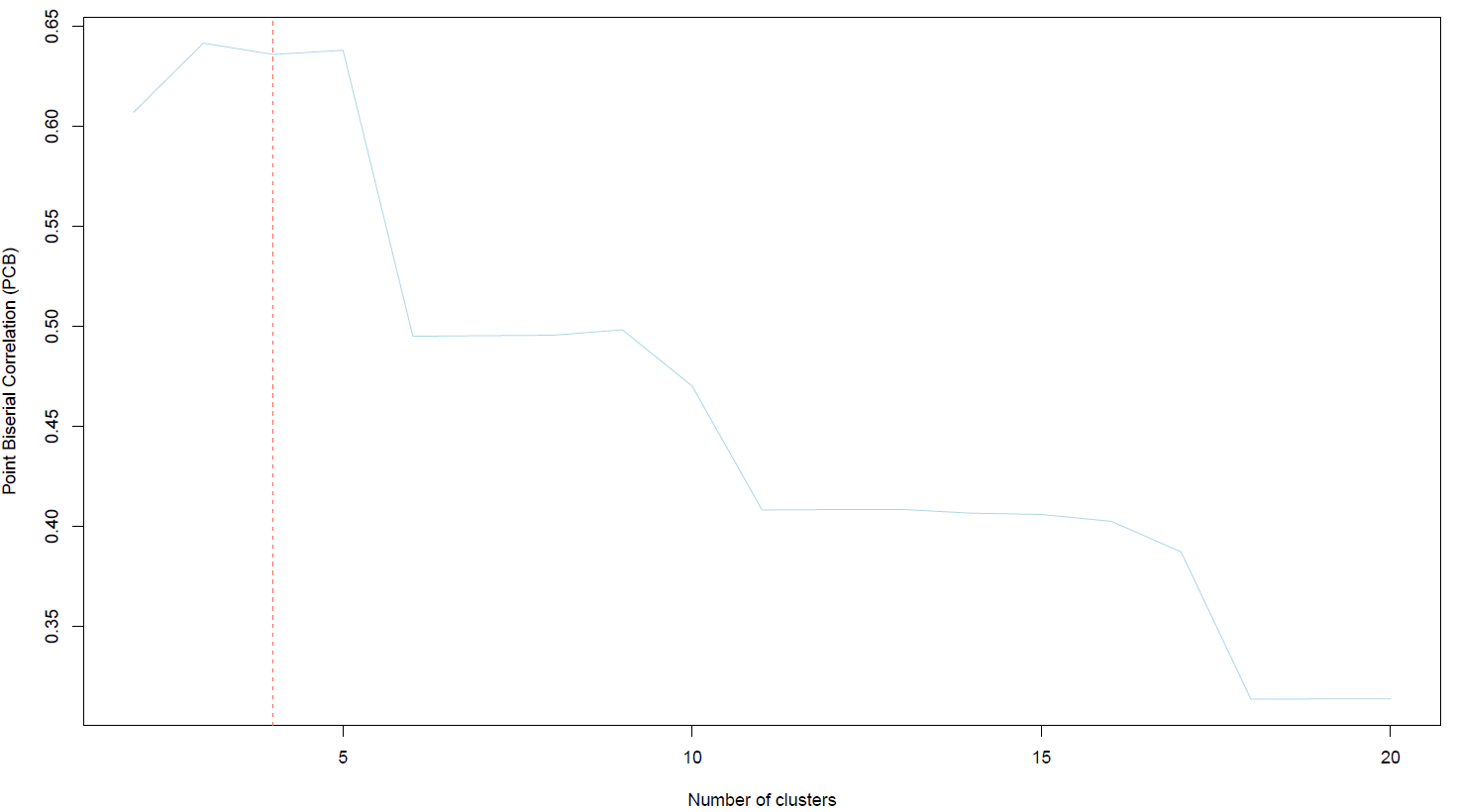

Supplement: S2 Fig — PBC was very similar for 3, 4 and 5 partitions. Therefore, we chose to build 4 clusters of patients (vertical dashed line). (PNG) [file pgph.0002821.s006.png]
