# Supplementary material for: Preventing iatrogenic HCV infection: A quantitative risk assessment based on observational data in an Egyptian hospital
Source: PLOS Glob Public Health. 2024 Feb 15;4(2):e0002821. doi: 10.1371/journal.pgph.0002821 (PMC10868760; doi:10.1371/journal.pgph.0002821)
Supplement: S1 Text — (DOCX) [file pgph.0002821.s007.docx]

**Text S1.** Mode calculation

The median of a PERT distribution can be approximated by $\frac{a+6b+c}{8}$ where $a$ and $c$ correspond to the minimum and maximum values of the risk and $b$ to the mode of the distribution. Knowing that $a = 1$ and $c = 9$·$2$, and assuming that the median is equal to 2·2%, we need to solve $\frac{a+6b+c}{8}=2\cdot2$ which gives $= 1\cdot23$ .
