# Supplementary material for: Preventing iatrogenic HCV infection: A quantitative risk assessment based on observational data in an Egyptian hospital
Source: PLOS Glob Public Health. 2024 Feb 15;4(2):e0002821. doi: 10.1371/journal.pgph.0002821 (PMC10868760; doi:10.1371/journal.pgph.0002821)
Supplement: Attachment — Submitted filename: Response to Reviewers.docx [file pgph.0002821.s008.docx]

Reviewer #1:

 1. Congratulations to the research team for their interesting study which addresses risk and how to prevent iatrogenic HCV infection in hospitals in Egypt.

2. The abstract should be more concise and informative, highlighting the main objectives, methods, results and implications of the study.

We thank the reviewer for this remark. Although the journal’s style does not allow for explicit headings within the abstract, we have now modified and restructured its content to make it more informative and clear to the reader by stating more precisely our objectives and results.

3. The introduction lacks detailed background information on HCV infection in Egypt (epi-data, morbidity and mortality, gaps in knowledge and why the objectives/aims of this study). If there is paucity of data on HCV infection in healthcare in Egypt, please indicate so. Lines 305-318 could be moved to the introduction.

We thank the reviewer for this useful comment and suggestion. We agree that the introduction section was lacking specific background information. As suggested we have moved lines 305-318 to the introduction and added more information on the Egyptian context in this section (L46 to L61) :

“While HCV prevalence varies widely between countries, Egypt has historically been the most affected country worldwide, with an HCV prevalence still over 10% in the adult population in the mid 2010’s [2]. The scaling-up of DAA treatments has temporarily yielded the hope that a large test-and-treat strategy could be sufficient to eliminate the epidemics [2]. In this context, Egypt launched an ambitious national treatment programme in 2014, followed by an intensive screening and treatment programme in 2018, with the objective of eliminating HCV within the country by 2021. In 2018 and 2019, almost 50 million Egyptian residents were screened (80% of the adult population), resulting in an HCV seroprevalence reduced to 4·6% in the adult population [3-4]. It is estimated that more than 90% of the HCV infected population were treated during this campaign, and the 2021 general prevalence was estimated at around 0.5%, thus achieving WHO elimination target. Despite these huge achievements, 20% of the adult population were not screened and treatment does not prevent for reinfections, which is especially problematic in specific population such as injectable drug users, whom could introduce HCV in hospitals. Hence, articulating primary HCV prevention, especially in healthcare settings [5], together with treatment and cure remains key to accelerate HCV elimination in the country.”

4. Methodology should include more information on data collection procedures. Lines 70-72 should be rephrased to ensure that the 15 and 10 wards are respectively referring to medical and surgical departments.

We again thank the reviewer for this remark. The mentioned sentence was rephrased as requested to clarify how many wards were in the internal medicine and surgery departments, and we have added more information on data collection in the Data and setting section (L86-100), as well as a reference to the original paper describing this study :

“*D*ata was collected as part of a prospective cohort study (ANRS 12320 IMMHoTHep project, “Investigative Mathematical Modeling of Hospital Transmission of Hepatitis C'') conducted over a 6-month period in 2017 [17]. This study focused on patients hospitalized in the internal medicine (organized in 15 wards) and surgery (organized in 10 wards) departments of the Ain Shams University Hospital in Cairo, Egypt. Five hundred hospitalized patients (aged more than 21) were included upon their admission to the hospital, either through the outpatient clinics or the emergency departments. Their demographic characteristics and medical history were collected through a structured questionnaire upon admission. Their HCV status upon admission was retrieved, and infections were confirmed by HCV-RNA detection. Patients’ individual trajectories were then followed up over the course of their entire hospitalizations: this included information on their geographical movements between departments and wards within the hospital and the invasive procedures they underwent within these locations. Procedures performed were aggregated into 15 groups following expert opinion (Table 1). Further information on the study is available in Anwar et al., (2021) [17].”

5. The findings and limitations were well presented. How does these limitations affect the accuracy and generalizability of the findings?

We agree with the reviewer that we did not discuss our findings’ generalizability enough. We have now added this information to the Discussion section (L306-313) :

“Our results might not be generalizable outside Egypt, as the epidemiology of HCV in this country is unique. In particular, in our database, most of the infected patients were older than non-infected ones and most of them had a history of anti-schistosomiasis treatment performed during the mass treatment campaign initiated by the government between 1950 and 1980, which led to a massive diffusion of HCV within the country [33]. While this may impact both the hotspots we identified within the hospital and the at-risk patient profiles we determined, the approach we propose to assess the HCV infection risk may still be extended to other settings and contexts beyond Egypt, provided the necessary data is available.”

6. The discussions of the findings is limited and should be expanded to include the major findings of the study as well as similar studies on HCV nosocomial infections to indicate similarities and differences in the findings and how they should be interpreted.

We thank the reviewer for this comment. The major findings are now more clearly stated in the Discussion section (L292-297) :

“While we estimated a low overall HCV infection risk, we found that some upon-admission patient characteristics were related to a higher risk: age, reason of hospitalization, and history of previous invasive procedures. We proposed a score to detect high risk patients upon their admission and assessed the effect of simulated interventions on the overall risk of HCV infection during hospitalization. Selecting patients using our score was always more effective than randomly selecting patients upon-admission.”

We agree that a comparison of our results to those of other published studies was lacking from the Discussion. A new section placing our work within the context of these earlier studies and comparing their findings with ours was added to the Discussion section (L314-L328) :

“To the best of our knowledge, only a few earlier studies have attempted to assess the individual risk of HCV infection for patients and healthcare workers within hospitals. Among these studies, only two described models investigating the patient-to-patient transmission risk using a probabilistic approach [14-15]. The first one [14] described the individual risk for patients hospitalized in a haemodialysis unit and the other one quantified the risk of bloodborne pathogen infection during an invasive procedure [15]. Although these studies used the HCV prevalence level within wards to estimate the HCV infection risk for hospitalized patients, none used detailed longitudinal data to investigate the individual infection risk during hospitalization. In addition, a few studies directly reported HCV incidence in hospitalized patients. However, all were focused on haemodialysis units [34-36]. Finally, as far as we know, no earlier study has investigated the impact on HCV infection risk of patient-based and ward-based interventions similar to those proposed in our work. Some studies investigated the effect of increased prevention and control in healthcare workers on the incidence of occupational exposure to bloodborne pathogens [37-38] but none seemed to focus on the effect of these prevention measures on the HCV incidence in patients.”

Reviewer #2:

"Preventing iatrogenic HCV infection: a quantitative risk assessment based on observational data in Egyptian Hospitals " is a very interesting research study to share. The authors were detailed in methodology and results. However, the discussion could not figure out at risk patients' profile before admission and the assessment of patients and ward focus strategies. the at risk patients in other studies were not compared with this study. Similarly, the assessment of patients and ward focus interventional strategies were not substantiated: were they the first of its kind reported or any comparison with other similar intervention. The limitations were fully stated and clear. This is an impressive manuscript if these observed parts were considered for discussion.

We thank the reviewer for these useful remarks. We added information about the characteristics of at-risk patient profiles in the discussion (L292-294) :

“While we estimated a low overall HCV infection risk, we found that some upon-admission patient characteristics were related to a higher risk: age, reason of hospitalization, and history of previous invasive procedures.”

As mentioned earlier, our results are not easily comparable to earlier published studies. In particular, to the best of our knowledge, the impact of interventions similar to those we simulate on the HCV infection risk in patients has never been assessed. We have now added this information to the Discussion section (L323-328) :

“Finally, as far as we know, no earlier study has investigated the impact on HCV infection risk of patient-based and ward-based interventions similar to those proposed in our work. Some studies investigated the effect of increased prevention and control in healthcare workers on the incidence of occupational exposure to bloodborne pathogens [37-38] but none seemed to focus on the effect of these prevention measures on the HCV incidence in patients.”
